# From Nature to Healing: Development and Evaluation of Topical Cream Loaded with Pine Tar for Cutaneous Wound Repair

**DOI:** 10.3390/pharmaceutics16070859

**Published:** 2024-06-26

**Authors:** Branislav Petrovic, Anica Petrovic, Katarina Bijelic, Dragana Stanisic, Slobodanka Mitrovic, Vladimir Jakovljevic, Sergej Bolevich, Ivana Glisovic Jovanovic, Jovana Bradic

**Affiliations:** 1 Department of Pharmacy, Faculty of Medical Sciences, University of Kragujevac, 69 Svetozara Markovica St., 34000 Kragujevac, Serbia; petrovicfmnkg@gmail.com; 2 Center of Excellence for Redox Balance Research in Cardiovascular and Metabolic Disorders, 69 Svetozara Markovica St., 34000 Kragujevac, Serbia; drvladakgbg@yahoo.com; 3 Department of Pharmacy, Faculty of Medicine, University of Novi Sad, Hajduk Veljkova 3, 21000 Novi Sad, Serbia; katarina.bijelic@mf.uns.ac.rs; 4 Center for Medical and Pharmaceutical Investigations and Quality Control, Faculty of Medicine, University of Novi Sad, Hajduk Veljkova 3, 21000 Novi Sad, Serbia; 5 Department of Dentistry, Faculty of Medical Sciences, University of Kragujevac, Svetozara Markovića 69, 34000 Kragujevac, Serbia; stanisic92@yahoo.com; 6 Department of Pathology, Faculty of Medical Sciences, University of Kragujevac, Svetozara Markovića 69, 34000 Kragujevac, Serbia; smitrovic1408@gmail.com; 7 Department of Physiology, Faculty of Medical Sciences, University of Kragujevac, Svetozara Markovića 69, 34000 Kragujevac, Serbia; 8 Department of Human Pathology, I.M. Sechenov First Moscow State Medical University, 119146 Moscow, Russia; bolevich2011@yandex.ru; 9 Orthopedic and Traumatology University Clinic, Clinical Center of Serbia, Dr Koste Todorovica 26, 11000 Belgrade, Serbia; ivanaglisa@gmail.com

**Keywords:** pine tar, wound healing, skin, oxidative stress, cream, rheology

## Abstract

Despite the numerous efforts to find an appropriate therapeutic modality, diabetic wounds remain a global unsolved problem. Therefore, our study aimed to develop a topical formulation loaded with pine tar and to investigate its wound-healing capacity. After phytochemical profiling of pine tar, an oil-in-water emulsion with 1% pine tar was prepared. The physical, chemical, and microbiological stability of prepared pine tar cream (PTC) was assessed during six months. Additionally, safety potential was examined in healthy rats, while wound-healing potential was accessed by creating excision wounds in diabetic rats. Diabetic animals were divided into four groups: untreated or topically treated with either the cream base, PTC, or silver sulfadiazine cream. Wound healing was monitored at the following time points (0, 7, 14, and 21 days) through macroscopic, biochemical, and histological examinations. Our PTC formula showed good physicochemical properties and remained stable and compatible for cutaneous application. PTC showed a remarkable increase in wound closure rate and led to attenuation of morphological alterations in skin samples. These findings were associated with significantly improved redox status and enhanced hydroxyproline levels in PTC relative to the untreated and cream base groups. Our results demonstrated that PTC might serve as a promising tool for the management of diabetic wounds.

## 1. Introduction

Pharmacoeconomic investigations revealed that over 8 million patients suffer from chronic wounds, significantly affecting their quality of life and escalating treatment costs and hospitalization rates, thereby imposing a substantial economic burden on the healthcare system [1,2]. Among chronic wounds, diabetic wounds stand out due to their compromised healing process, susceptibility to infections, prolonged treatment duration, and associated pain. Unfortunately, optimal wound dressings for diabetic wounds remain elusive [1,2].

Currently, available therapeutic modalities in the management of chronic wounds involve surgical debridement, wound dressings, antibiotic use, hyperbaric oxygen, etc. [3]. Evidence suggests that patients with chronic wounds who receive antibiotics for a long time are at higher risk of developing antimicrobial resistance. Furthermore, this can consequently prolong treatment duration, decrease effectiveness, increase medical costs, and lead to a high mortality rate [1,4]. Additionally, antibiotic overuse contributes to environmental contamination, exacerbating ecological concerns [5]. Besides antibiotics, therapy of diabetic wounds is based on topical or oral anti-inflammatory drugs, which can cause different skin reactions (redness, itching, edema, etc.) and resistance. Moreover, wound dressings play an important role in wound management, and they are often used in combination with antibiotics to foster efficient inflammation and proliferation [6]. Traditional wound care dressings consist mainly of low-technology gauze-based dressings such as woven and non-woven sponges, conforming bandages, and non-adherent bandages, which prevent infection while being almost ineffective for wound healing. Also, different kinds of wound dressings composed of synthetic materials (nylon, polyurethane, silicone, polymers) are not suitable for highly exuding wounds [7,8].

In light of these challenges, many scientists are searching for naturally based formulations that exert antimicrobial, anti-inflammatory, antioxidant, and wound-healing properties as safer and more sustainable alternatives to antibiotics. Since ancient times, various plant species have been used to treat different kinds of conditions and diseases. Herbal medicine emphasizes the importance of biologically active compounds from various plants and their pharmacological effects, especially in treating wounds [9]. Literature supports the beneficial effects of *Pinus* species in wound healing across different experimental models [10,11,12,13].

Over two centuries, pine tar, a brown or black viscous liquid, has been produced through the utilization of the pyrolytic process known as dry or destructive distillation of different *Pinus* species (such as *Pinus nigra*, *Pinus brutia*, *Pinus pinea*, *Pinus halepensis Mill*, *Pinus sylvestris* L., etc.). The main components of pine tar include terpenes, phenols, resins (fatty acids), and other components such as toluene and xylene [14]. Despite its historical use in dermatological conditions (psoriasis, eczema, seborrheic dermatitis, dry and wounded skin), the precise mechanisms underlying its therapeutic effects remain incompletely understood [15,16,17,18]. Therefore, our investigation was the first to formulate and characterize an herbal cream with pine tar and assess its stability, safety, and efficacy in the treatment of diabetic wounds in rats.

## 2. Materials and Methods

### 2.1. Chemicals

Substances such as dichloromethane (POCh, Gliwice, Poland), N,O-Bis(trimethylsilyl)trifluoroacetamide—BSTFA (Merck, Darmstadt, Germany), and pyridine (Centrohem, Stara Pazova, Serbia) were used for chromatography. The preparation of creams was achieved with stearic acid, cetyl alcohol, cetearyl alcohol, polysorbate 80, sweet almond oil, glycerin, distilled water, triethanolamine, citric acid, and phenoxyethanol which were purchased from Unichempharm, Cacak, Serbia. Standard buffer solutions for measuring pH and electrical conductivity were purchased at Mettler Toledo, Columbus, OH, USA. Xylazine, ketamine, and streptozotocin were provided by Merck, Darmstadt, Germany. Reagents for determination of hydroxyproline content (HCl, chloramine T, Ehrlich reagent) and reagents for systemic redox state parameters (sulfanilic acid, N-(1-naphthyl)-ethylenediamine dihydrochloride, ammonium chloride, borax, 85% ortho-phosphoric acid, sodium nitrite, 2-thiobarbituric acid, 28% trichloroacetic acid, sodium hydroxide, tris(hydroxymethyl)aminomethane, 37% hydrochloric acid, nitro-tetrazolium blue chloride, gelatin, potassium hydrogen phosphate dehydrate, potassium dihydrogen phosphate dehydrate, sodium chloride, hydrogen peroxide, D(+)-glucose monohydrate, phenol red, horseradish peroxidase, distilled water, ethanol, disodium phosphate, sodium carbonate, ethylenediaminetetraacetic acid, epinephrine, 5,5′-dithio-bis (2-nitrobenzoic acid), ethylenediaminetetraacetic acid, metaphosphoric acid, glutathione) were purchased from Sigma-Aldrich, Darmstadt, Germany. Substances for histological analysis (formalin, isopropyl alcohol, paraffin, hematoxylin–eosin solution) were provided from Sigma-Aldrich, Darmstadt, Germany.

### 2.2. Pine Tar Production

The plant material utilized in this research comprises stumps of *Pinus nigra*, collected from mountain Mokra Gora in April 2023. Upon collection, bark samples were chopped and subjected to a process of dry distillation under extreme heat in a kiln to extract pine tar. Subsequently, the samples were transported to the laboratory and stored in bottles at room temperature.

### 2.3. Chemical Composition of Pine Tar

First, 30 mg of pine tar sample was dissolved in 15 mL of dichloromethane using an ultrasonic bath for 30 min. An aliquot of 1 mL of the dissolved sample was then evaporated to dryness in a stream of nitrogen and 250 µL of derivatization reagent (N,O-Bis(trimethylsilyl)trifluoroacetamide—BSTFA) and 25 µL of pyridine were added. Derivatization was performed for 45 min at 60 °C. After derivatization, the mixture was evaporated to dryness. The dry residue was dissolved in 500 µL dichloromethane and further analyzed by a gas chromatography system (7890B GC System, 5997A MSD; Agilent Technologies, Waldbronn, Germany) with parallel MS and FID detection, using an HP-5MS (30m × 0.250mm × 0.25 μm; Agilent Technologies) column. The previously described chromatographic separation of compounds of interest [19] was somewhat modified. The initial oven temperature was 100 °C (1 min); after that, the temperature was raised to 325 °C (5 °C/min) and then maintained for 10 min. The temperature of the injector was 270 °C, and the split mode was used at 1:10. The MS was operated in scan mode (m/z from 50 to 700). The FID temperature was set at 320 °C. The flow of helium was constant at −1 mL/min, and the transfer line temperature was 300 °C. The identification of compounds present in pine tar was based on the comparison of relative retention indices (RI) as well as the comparison of mass spectra with spectra from the NIST v14 database.

### 2.4. Preparation of Cream Base and Pine Tar Formulation

The cream base was made by precisely measuring and mixing oil phase components (stearic acid, cetyl alcohol, cetearyl alcohol, polysorbate 80, and sweet almond oil) and the aqueous phase (glycerin, distilled water, 10% TEA) separately withheating up to 70–80 °C. After heating both phases, the oil phase was added to the aqueous phase with continuous stirring on a laboratory stirrer RV16 basic (IKA^®^VERKE, Staufen im Breisgau, Germany). The emulsion was cooled down, and a preservative (phenoxyethanol) and pH adjuster (citric acid) were added. To create a pine tar formulation, 1% pine tar was added in the cooling down stage in the above-mentioned base by the levigation method [20]. All ingredients of both formulations are presented in Table 1.

### 2.5. Physical Evaluation of Topical Formulations

#### 2.5.1. Organoleptic Characteristics

The prepared samples underwent organoleptic analysis, including visual observation, to evaluate characteristics such as color, odor, homogeneity, and the presence of phase separation in the creams [20].

#### 2.5.2. pH Values

The pH values of the prepared creams were determined using a digital pH meter (Mettler Toledo, Columbus, OH, USA) calibrated with standard buffer solutions at pH 4.0, 7.0, and 9.0 prior to measurements. The pH measurements were conducted in triplicate [21].

#### 2.5.3. Electrical Conductivity Values

The conductivity meter (Eutech CON 700, Thermo Fisher Scientific, Shanghai, China) was calibrated, and the electrical conductivity (σ) of samples was measured at room temperature. All measurements were performed in triplicate [22].

#### 2.5.4. Centrifugation Test

A centrifugation test was performed directly upon cream preparation to evaluate changes such as phase separation. Samples were centrifuged in a laboratory centrifuge (Hettich Mikro 120, Andreas Hettich GmbH & Co. KG, Tuttlingen, Germany) at 3000 rpm at room temperature for 30 min. Samples were considered to be stable if they remained homogenous after centrifugation [23].

#### 2.5.5. Rheological Characterization of Cream Formulations

The viscosity curves of pine tar cream and base cream were measured with an Anton Paar MCR 102e Rheometer (Anton Paar, Graz, Austria) equipped with a peltier temperature device. Logarithmic shear rate ramps from 0.1 s^−1^ to 1000 s^−1^ at constant temperatures of 25 ± 0.1 °C and 37 ± 0.1 °C were performed. In order to avoid transition effects at low shear rates and expulsion from the measuring gap at high shear rates, the duration of the measuring point was logarithmically reduced from 10 s to 1 s. A plate–plate PP25 measuring system was used with a 1 mm gap [24]. Temperature equilibration was carried out for 3 min prior to starting the measurements. Rheocompass version 1.31 software (Anton Paar, Graz, Austria) was used to control the device and acquire measured data.

#### 2.5.6. Assessment of Long-Term Stability of the Pine Tar-Loaded Formulations

Long-term stability analyses (organoleptic characteristics, pH values, electrical conductivity, and microbiological tests) were performed for topical formulations that were stored in an incubator (ICT-R 52 FALC, Falc Instruments, Treviglio, BG, Italy) at different temperatures such as 4 ± 2 °C, 25 ± 2 °C, and 40 ± 2 °C (accelerated aging). All parameters were observed at various time intervals over six months. The measurements were taken 24 h, 90, and 180 days after preparation [25].

Both cream formulations were tested according to the following standards: SRPS EN ISO 21149: 2017, SRPS EN ISO 16212: 2017, SRPS EN ISO 18416:2016, SRPS EN ISO 21150:2016, SRPS EN ISO 22717:2016, SRPS EN ISO 22718:2016 [26,27,28,29,30,31] used to detect the specified microorganisms. The presence of noteworthy pathogens like *Escherichia coli*, *Pseudomonas aeruginosa*, *Staphylococcus aureus*, and *Candida albicans* were identified. On the other hand, we had the ability to enumerate the overall aerobic mesophilic bacteria, including both the total aerobic microbial count and the total yeast and mold count [32]. Long-term analyses were performed after 24 h, 90, and 180 days of preparation during the above-mentioned storage conditions.

### 2.6. In Vivo Investigation

#### 2.6.1. Ethical Statement

The first part of the investigation was conducted at the Center for Preclinical and Functional Investigations of the Faculty of Medical Sciences, University of Kragujevac, Serbia, according to the European Directive for Protection of the Vertebrate Animals used for Experimental and Other Scientific Purposes 86/609/EES and the principles of Good Laboratory Practice. The Faculty’s Ethical Committee for the Welfare of Laboratory Animals approved the experimental protocol (number 01–12408).

#### 2.6.2. Acute Dermal Irritation of Cream Formulations

The dermal irritation test was conducted according to guidelines OECD 404 on healthy *Wistar albino* rats (number: 6; sex: male: b.w. 200–250 g). Twenty-four hours before experiments, the rat’s fur was removed, and cream formulations were applied to the precisely marked dorsal area and protected with a gauze patch. Rats were treated with (1) pine tar cream—PTC (*n* = 3) and (2) cream base—CB (*n* = 3).

The tested formulations were applied topically in an amount of 500 mg to the shaved area. After that, the rats were placed in individual cages. The animals were observed with special attention during the first 4 h after administration of the preparation, after which they were observed once a day for a period of 14 days. The rats were observed and recorded if mortality or any signs of toxicity, such as tremors, salivation, convulsions, or diarrhea, occurred. The occurrence of adverse effects on the skin, such as edema and erythema, was also monitored based on the scoring system, with 0 indicating the absence of erythema/edema, 1 indicating very inappreciable edema or erythema, 2 indicating small edema with raised skin at the edges of the area, 3 indicating moderate to severe erythema or edema, and 4 indicating severe erythema/edema [10].

#### 2.6.3. Wound Healing Capacity

Forty healthy *Wistar albino* rats (250–300 g) were procured from the Military Medical Academy, Belgrade, Serbia, and housed in clean cages under the following conditions: 12 h day/12 h dark, at an ambient temperature of 22 ± 2 °C, with unlimited access to water and food. After whole night starvation, all rats received 50 mg/kg streptozotocin (STZ) intraperitoneally (i.p.). A portable glucometer (Accu-Check^®,^ Roche, Basel, Switzerland) was used to measure blood glucose. Values of glucose > 11.1 mmol/L were considered significant for confirming diabetes mellitus [33]. Animals were randomly assigned into four groups of 10 rats each:

(1) NC (negative control, untreated group);
(2) PC (positive control, wounds treated with 1% silver sulfadiazine);
(3) CB (wounds treated with cream base);
(4) PTC (wounds treated with 1% pine tar cream).

The experimental protocol started with i.p. administration of anesthesia (xylazine (10 mg/kg) and ketamine (5 mg/kg)). After first shaving the dorsal area of animals and cleaning it with 70% ethanol, excision wounds (2 × 2 cm sized, 2 mm depth) were created by precisely using a scalpel [34]. Animals were photographed, and prepared creams were applied in an amount of 500 mg per day for 3 weeks. On the 7th, 14th, and 21st day of treatment, selected rats were injected with a mixture of ketamine (10 mg/kg) and xylazine (5 mg/kg) to collect blood and skin samples for estimation of oxidative stress parameters, determination of hydroxyproline content, and histopathological analysis, respectively.

##### Wound Contraction Estimation

The wound area was monitored from day 0 until the end of 21 days of wound creation by photographing wounds and using Fiji version of Image J software 2.9.0 (National Institutes of Health, Bethesda, MD, USA). The degree of healing was calculated on the 7th, 14th, and 21st days and expressed as wound contraction, which is presented as a percentage of wound contraction/closure using the formula below [35].

$$\%Wound\;contraction=\left(\frac{Healed\;area}{Intial\;wound\;area}\right)\times 100$$

* Healed area = Initial wound area – Wound area on a specific day

##### Hydroxyproline Content Estimation

Hydroxyproline content was measured to determine the total collagen content in the tissue according to a previously established protocol [10]. Absorbance was recorded at 557 nm via a spectrophotometer (Shimadzu UV-1800, Shimadzu, Tokyo, Japan), and the content of hydroxyproline was expressed as μg/mg dry tissue weight after plotting the absorbance against a pure hydroxyproline calibration curve [35].

##### Determination of Pro-Oxidative and Antioxidant Defense System Markers

At the time of animal sacrifice, parameters of the systemic redox state were obtained from jugular vein blood samples, following our previously established protocol [10]. The supernatant from centrifuged blood samples was utilized to determine the concentrations of oxidative stress parameters, hydrogen peroxide (H_2_O_2_), index of lipid peroxidation (measured as TBARS), including nitrites (NO_2_^−^), and superoxide anion radical (O_2_^−^). Moreover, erythrocyte lysate was used for antioxidative system markers determination: the activity of antioxidant enzymes such as catalase (CAT) and superoxide dismutase (SOD) and the level of reduced glutathione (GSH).

### 2.7. Histology of Wound Tissue Samples

Following animal sacrifice, skin samples from the healed area were collected and fixed in 10% buffered formalin at 4 °C for a minimum of 24 h to facilitate histological examination. Subsequently, the fixed tissue sections underwent dehydration in solutions with increasing concentrations of isopropyl alcohol (70–100%). They were then embedded in paraffin under vacuum and sectioned at a thickness of 5 µm using a rotating microtome (Leica, Wetzlar, Germany). The tissue sections were stained with hematoxylin–eosin (H&E) for further analysis.

### 2.8. Statistics

IBM SPSS Statistics for Windows version 23.0 (IBM Corporation, Armonik, NY, USA) was used for statistical analysis via one-way ANOVA, using Tukey’s multiple comparison post hoc tests. All data were expressed as mean ± standard deviation. Values of *p* < 0.05 were considered to be statistically significant.

## 3. Results and Discussion

### 3.1. Chemical Composition of Pine Tar

Thirty-two components were identified in pine tar, representing 95.50% of the total tar composition. The most abundant compounds detected in pine tar were methyl dehydroabietate (22.44%), dehydroabietic acid (14.59%), and reten (10.08%). All detected chemical compounds are presented in Table 2.

According to literature data, the chemical composition of pine tar is complex, constituting mainly aromatic hydrocarbons, tar acids, and bases. In addition, chemical composition depends on various factors, such as the species of pine trees, production method, and purification process. In the earlier study, the main constituents of pine tar from the *Pinus sylvestris* tree were dehydro abietic acid and abietic acid, which is similar to our investigation [36]. To our best knowledge, this is the first study revealing chemical constituents of tar from *Pinus nigra*.

### 3.2. Physicochemical Characterization of Pine Tar-Based Formulation

#### 3.2.1. Organoleptic Characteristics and Physical Appearance

The results obtained for the organoleptic characteristics of cream formulations during six months at three different temperatures are presented in Table 3. CB exhibited a white color and was odorless, while the addition of pine tar transformed pine tar cream (PTC) into a brown color with a characteristic odor for pine tar. Both formulations had semi-solid consistency and were homogenous. Analyzing the stability of formulations at different temperatures, we did not notice changes in organoleptic parameters. Furthermore, during a period of six months at different storage conditions, both cream formulations remained stable, showing good properties without phase separation.

The ingredients used in the formulations play a crucial role in achieving satisfactory organoleptic properties and ensuring the stability of the product over time. For instance, stearic acid, as an emollient, thickening agent, and emulsifier, plays a pivotal role in topical formulations since it can enhance homogeneity, help maintain their integrity, and prevent phase separation [37]. All aforementioned properties of cream formulations are essential for commercial products and can significantly influence consumer decisions [38].

#### 3.2.2. pH Values and Electrical Conductivity

The pH values of both the cream base and the pine tar-loaded formulation (CB and PTC) are shown in Table 4. The pH of the cream base formulation was in the range of 5.26 ± 0.04 to 5.72 ± 0.03, while the pine tar-based formulation was in the range of 6.01 ± 0.04 to 6.72 ± 0.03 during a six-month storage period at different temperatures. Although we noticed a slight decline in pH values over the observed period at different temperatures, values did not change remarkably. Moreover, the pH values of the examined formulations were within the proposed range for skin application. The main reason for measuring and adjusting the pH value in the formulation is to avoid irritation, dryness, redness, inflammation, and damage to the skin [39,40]. Furthermore, the addition of pine tar in the formulation slightly enhanced pH values but was still in an adequate range for skin application. These pH values of cream formulations were as expected for this stearic type of cream.

Table 4 displays the electrical conductivity values of the formulations CB and PTC under different storage conditions over 24 weeks. The range of electrical conductivity for CB was from 60.2 ± 0.07 µS/cm to 77.6 ± 0.02 µS/cm, while PTC was from 67.2 ± 0.04 µS/cm to 79.1 ± 0.01 µS/cm. Electrical conductivity values measured in both formulations did not change remarkably and indicated that our formulations were the O/W type of emulsion. In addition, a higher storage temperature (40 ± 2 °C) slightly increased electrical conductivity values, likely due to a larger amount of free water in comparison to lower storage temperatures (4 ± 2 °C). Water, being a polar solvent, can enhance the ionization of certain substances within the formulation, leading to increased electrical conductivity [41]. However, these values decreased during the storage time. There are many factors affecting the conductivity values of the formulations, such as their composition, encompassing components such as water, emollients, thickeners, and other ingredients [42]. Importantly, loading pine tar to a formulation did not affect this parameter.

#### 3.2.3. Centrifugation Test

**Figure 1:**
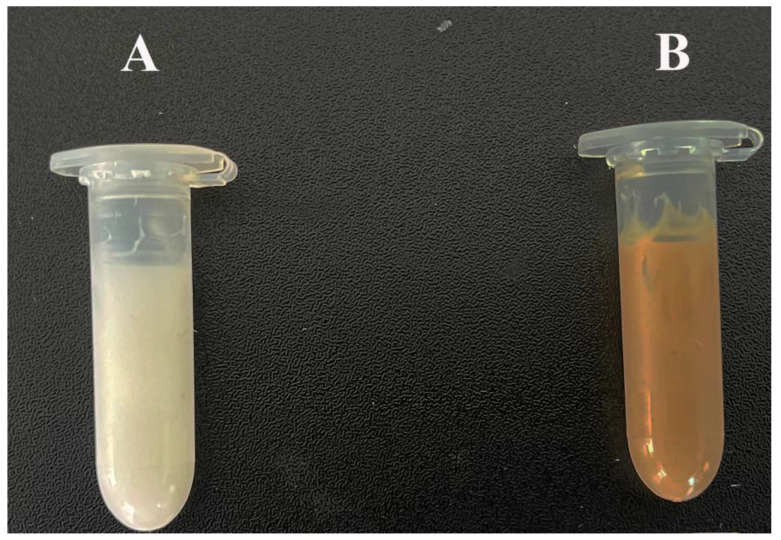
Photograph of cream base (**A**) and PTC (**B**) formulations after the centrifugation test.

After centrifugation of both cream formulations (CB and PTC), we did not observe decomposition, separation, or precipitation of the components, as depicted in Figure 1.

#### 3.2.4. Rheological Characteristics of the Formulations

**Figure 2:**
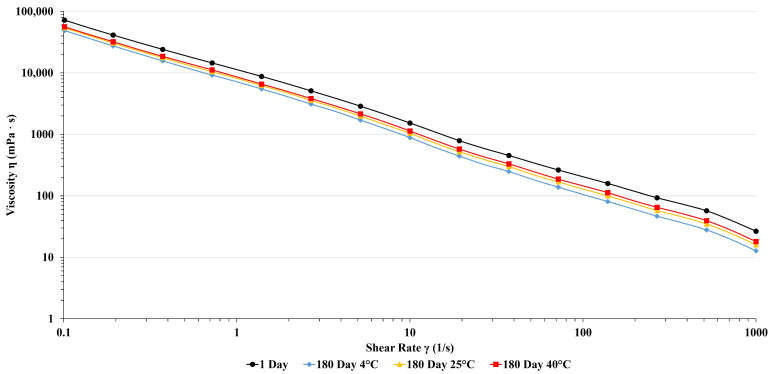
Viscosity curves of PTC after 24 h and six months of storage at different temperatures measured at 25 °C.

**Figure 3:**
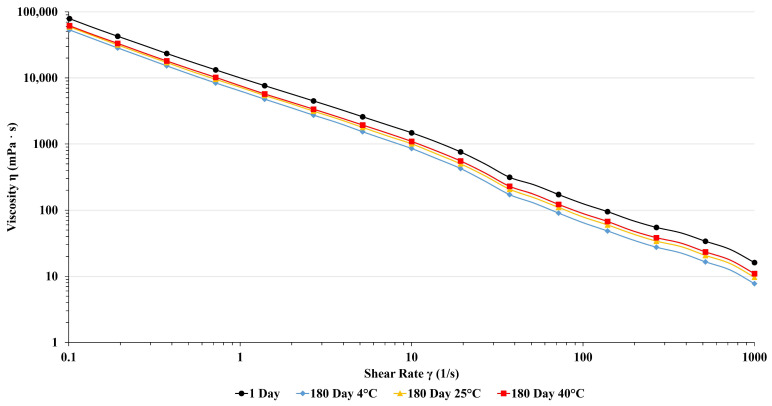
Viscosity curves of PTC after 24 h and six months of storage at different temperatures measured at 37 °C.

The rheological properties of dermal products provide valuable insight into the physicochemical stability of semi-solid products when various forces are used. Moreover, assessing the rheological profile of the topically applied formulation can reveal their ability to adhere to mucous membranes and their influence on the release rate of active ingredients. To investigate product quality and performance on the skin, the viscosity of PTC changes on different shear rates at temperatures (25 °C and 37 °C) was examined. Results are presented in Figure 2 and Figure 3.

Viscosity curves of pine tar cream and base cream show typical shear thinning profiles of emulsions. Viscosity values at 0.1 s^−1^ shear rate, which simulates the state at rest, are in the range of between 50 Pa*s and 70 Pa·s, and there is no significant difference between measured values at 25 °C and 37 °C. This indicates a strong network of forces built within samples, which will provide good stability. With increasing shear rate, viscosity values abruptly decrease, which is usual for semi-solid preparations. During shearing, shear stress overcomes a network of forces within the sample, and the sample passes from elastic deformation through the yield region into viscous flow. At higher shear rates from 100 s^−1^ to 1000 s^−1^, which simulates spreading, viscosity values are low (around 10 mPa·s). This will reduce friction and allow easy application of cream on the skin. Moreover, viscosities of accelerated aging samples, although a bit lower than the starting composition, still have similar values. Our results demonstrated that storing PTC formulation at different temperatures over six months did not alter its non-Newtonian pseudoplastic rheological behavior. The viscosity of PTC decreased as the shear rate increased. Importantly, performing these experiments at different temperatures did not affect the viscosity of the formulations. In light of the above-mentioned results, the behavior of the PTC formulation suggests easy application, spreadability, and sustained adherence to the skin [43].

#### 3.2.5. Microbiological Stability Test

Ensuring the robust microbiological integrity of semi-solid aqueous products is just as crucial as maintaining their physical stability during storage. Microorganisms such as bacteria, mold, and yeast wield significant influence over the stability of dermal creams [44]. The proliferation of microbes and the production of their metabolic byproducts can lead to changes in pH values, phase separation, or aggregation within a cream, consequently affecting its texture, appearance, and odor. Additionally, certain microorganisms can produce toxins and contaminate products that may cause skin irritations to the consumer [45]. Therefore, microbial testing is necessary and crucial to promptly detect microbiological contamination. In light of these facts, major skin pathogens such as *E. coli*, *P. aeruginosa, S. aureus,* and *C. albicans* were investigated in CB and PTC formulations. During the storage period, both prepared semi-solid formulations (CB and PTC) meet all ISO standards and requirements for microbiological safety. Specifically, we observed the absence of *E. coli*, *P. aeruginosa*, *S. aureus,* and *C. albicans*, while a number of aerobic mesophilic bacteria, yeast, and molds were lower than 1 cfu/1g. This success can be attributed to the presence of a natural preservative (phenoxiethanol), which provided good microbiological stability over six months. Furthermore, it has been previously demonstrated that pine tar exhibits antibacterial activity [15].

### 3.3. In Vivo Investigation on Animal Model

#### 3.3.1. Acute Dermal Irritation of Topical Formulations

Application of CB and PTC did not show any clinical signs of acute dermal toxicity such as erythema, edema, and other clinical toxicity manifestations such as pruritus, erythema, and inflammation during a 14-day observation. During the monitored period, we did not observe behavioral changes.

#### 3.3.2. Measurement of Wound Contraction

**Figure 4:**
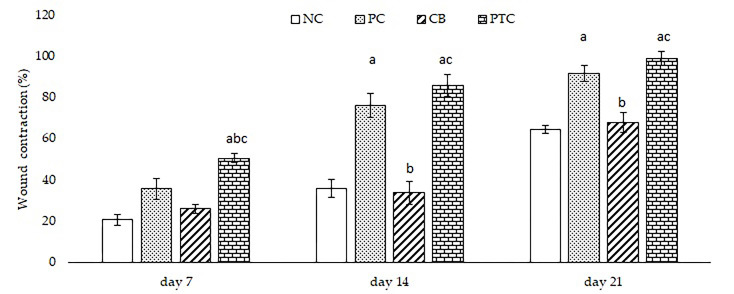
Effect of examined formulations on wound contraction at three time points. Values are mean ± standard deviation. ^a^ *p* < 0.05 compared to NC group; ^b^ *p* < 0.05 compared to PC group; ^c^ *p* < 0.05 compared to CB group; NC—negative control; PC—positive control; CB—cream base; PTC—pine tar cream.

**Figure 5:**
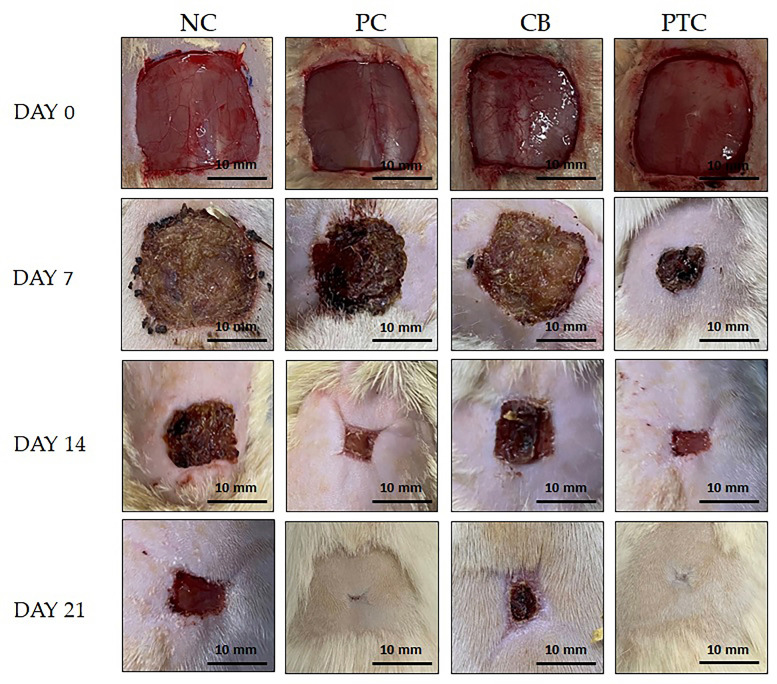
Photographed wound area during three weeks of application using an excision wound model, with observations recorded on days 0, 7, 14, and 21. NC—negative control; PC—positive control; CB—cream base; PTC—pine tar cream.

After achieving positive results in stability and safety experiments with PTC, our focus turned to examining their impact on wound healing in diabetic rats. In order to ensure reliable results, PTC was compared with a commercially available cream and cream base. To our knowledge, this is the first study to investigate and confirm the healing properties of pine tar incorporated in cream as a semi-solid formulation. The initial parameter that we measured was wound contraction, expressed as the percentage reduction in the wound area, which was assessed across weeks, as illustrated in Figure 4 and Figure 5. Following seven days of topical application of 1% pine tar formulation, we noticed a significant increase in wound contraction compared to the untreated rats (NC) group and groups treated with 1% silver sulfadiazine and cream base (PC and CB groups). As illustrated in Figure 4, the reduction of wound area was notably significant after 14 and 21 days in the PTC group relative to the NC and CB groups. Moreover, topical treatment with 1% silver sulfadiazine showed a significant increase in wound contraction compared to the untreated group and the group treated with a cream base. After 21 days of dermal treatment of diabetic wounds, the highest percentage of wound contraction was observed in the PTC group, 98.78%, while standard cream exhibited a maximum rate of wound contraction of 91.58%.

The heightened contractile properties induced by pine tar formulation are likely attributed to the presence of chemical compounds such as dehydroabietic, abietic acid, and its derivates (methyl dehydroabiatate). Literature data suggest that the most dominant compound methyl dehydroabiatate has already demonstrated antibacterial and antifungal properties [46]. Moreover, abietic acid may enhance wound healing by accelerating angiogenesis [47] and antioxidant activity [48], while dehydroabietic acid exerts antimicrobial activity against *C. albicans*, *S. aureus*, and *S. epidermidis* [49,50] alongside anti-inflammatory properties [51]. Research into the effects of terpenes and phenols on tissue regeneration and wound healing is ongoing, but existing studies suggest their potential as therapeutic agents in promoting wound healing and tissue repair [52].

#### 3.3.3. Evaluation of Hydroxyproline Content

**Figure 6:**
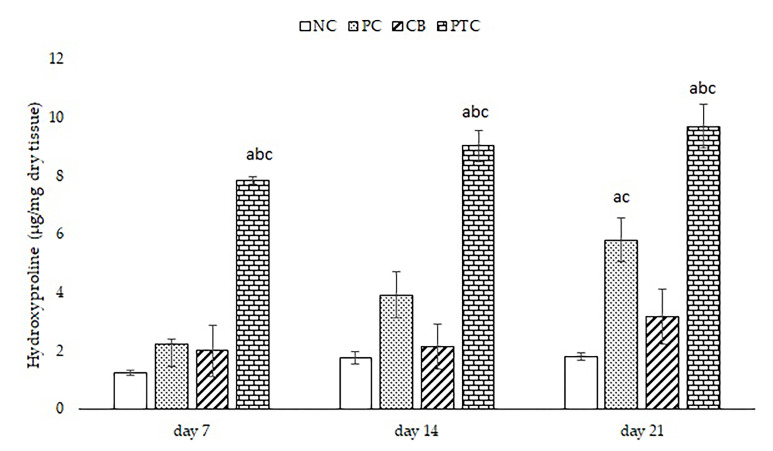
Effects of topical formulations on hydroxyproline content after 7, 14, and 21 days of administration. ^a^ *p* < 0.05 compared to NC group; ^b^ *p* < 0.05 compared to PC group; ^c^ *p* < 0.05 compared to CB group; NC—negative control; PC—positive control; CB—cream base; PTC—pine tar cream.

Considering the pivotal role of collagen in skin repair, serving as an essential component in the formation of new tissue and the restoration of structural integrity following injury or damage, we determined the level of hydroxyproline as a key building block for collagen. Hydroxyproline contributes to collagen stability, strength, and function in supporting and repairing tissues throughout the body [53]. The hydroxyproline content in the wound tissues of the diabetic rats is depicted in Figure 6. The group treated topically with PTC exhibited the highest level of hydroxyproline at all time points. Importantly, on the 21st-day post-wounding, the hydroxyproline level in the PTC group was nearly five times higher compared to the untreated group, while it was significantly higher in relation to the PC group. The same upward trend was observed at 7 and 14 days of treatment in the PTC group compared to the NC group, indicating the speed of effectiveness of pine tar formulation. Given the fact that phenols, as the most dominant compounds of pine tar, have a pronounced effect on collagen crosslinks in the body, an enhanced level of hydroxyproline was expected [54]. Earlier reports indicate that phenolic hydroxyl groups exhibit high reactivity with collagen, and the augmentation in both molecular complexity and weight of polyphenols significantly enhances their crosslinking capacity [55]. Furthermore, our previous study showed that the application of *P. sibirica* essential oil formulations increased hydroxyproline content, which can be attributed to terpenes that are abundant compounds in pine tar [10]. This supports the idea that the presence of terpenes in pine tar may contribute to the observed increase in hydroxyproline levels in the current study.

#### 3.3.4. Systemic Redox Status Markers

Our investigation placed special emphasis on revealing the role of oxidative stress in the wound-healing process. Oxidative stress occurs when the body produces an abundance of pro-oxidants, such as reactive oxygen and nitrogen species, while antioxidant levels are reduced. This imbalance can trigger cellular damage and consequently can induce a range of diseases. Furthermore, we aimed to elucidate if our cutaneous formulation may regulate the level of antioxidants via direct neutralization of free radicals. Indeed, the reduced production of reactive oxygen species (ROS) and/or enhanced antioxidant activity may facilitate faster wound repair and improve disturbed wound-healing processes [56].

**Figure 7:**
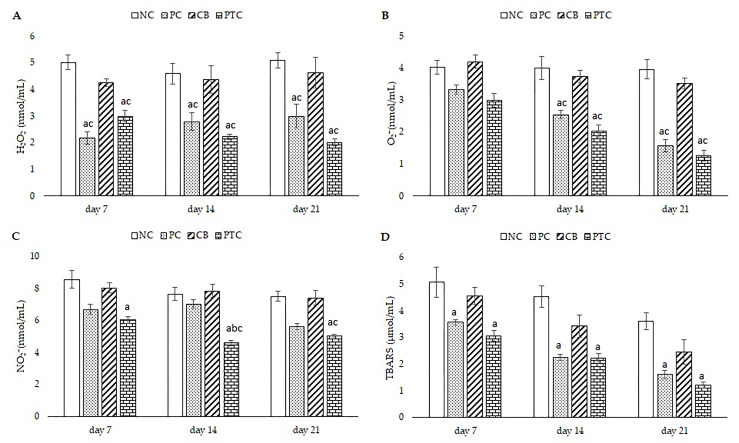
Effect of topical formulations on the pro-oxidative markers over 21 days: (**A**) H_2_O_2_; (**B**) O_2_^−^; (**C**) NO_2_^−^; (**D**) TBARS. The results are presented as the mean ± standard deviation. ^a^ Statistical significance at *p* < 0.05 in relation to NC group; ^b^ statistical significance at *p* < 0.05 in relation to PC group; ^c^ statistical significance at *p* < 0.05 in relation to CB group. NC—negative control; PC—positive control; CB—cream base; PTC—pine tar cream.

Topical treatment with 1% pine tar cream or 1% silver sulfadiazine cream significantly reduced H_2_O_2_ concentration in comparison to NC and CB groups. This reduction persisted over time, where a significant reduction was observed after 7, 14, and 21 days of treatment. Moreover, PTC significantly lowered the levels of O_2_^−^ after two and three weeks of dermal application relative to the NC and CB groups. A similar result was observed in rats treated with commercial cream (PC group). Interestingly, levels of NO_2_^−^ were remarkably decreased after 14 and 21 days of treatment compared to the NC and CB groups. Levels of lipid peroxidation, measured as TBARS, were significantly lower in rats treated with PTC or PC relative to the NC group. All results are presented in Figure 7. Our analyses suggest that enhanced production of pro-oxidants such as H_2_O_2_, O_2_^−^, and TBARS in untreated (NC) and vehicle groups (CB) likely contributed to prolonging the wound-healing process. Topical treatment with a cream base did not reduce the level of any measured pro-oxidants. These results are particularly interesting given that conditions such as diabetes mellitus lead to accelerated production of reactive oxygen and nitrogen species, which significantly hinder the wound-healing process [4]. Our previous findings demonstrated that terpenes from *P. sibirica* essential oil significantly reduced systemic pro-oxidative markers [10]. Literature data indicate that terpenes as aromatic compounds can decrease oxidative stress via reduction in lipid peroxidation, ROS formation, NO release, and suppressing NF-κB and MAPK signaling pathways [57]. Since the chemical composition of pine tar also comprises phenols, it was expected that the combination of terpenes and phenols would have a synergistic effect on decreasing oxidative damage. The primary phenol detected in pine tar preparations is eugenol, which contains phenolic groups that have the ability to reduce ROS by donating electrons or hydrogen atoms. This action breaks the chain reaction of oxidative damage, thereby reducing oxidative stress [58].

**Figure 8:**
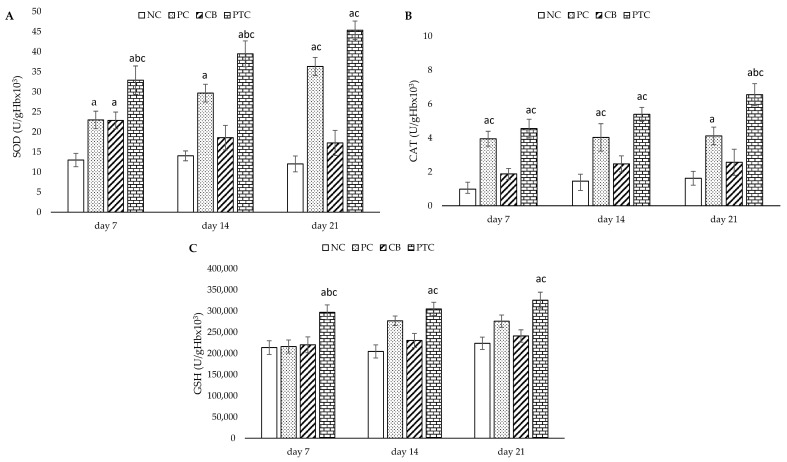
Effect of topical formulations on antioxidative defense system parameters: (**A**) CAT; (**B**) SOD; (**C**) GSH. The results are presented as the mean ± standard deviation. ^a^ Statistical significance at *p* < 0.05 in relation to NC group; ^b^ statistical significance at *p* < 0.05 in relation to PC group; ^c^ statistical significance at *p* < 0.05 in relation to CB group. NC—negative control; PC—positive control; CB—cream base; PTC—pine tar cream.

On the other hand, cutaneous treatment with PTC remarkably enhanced the activity of antioxidant enzymes SOD, CAT, and GSH compared to the NC and CB groups. This increase showed a consistent upward trend over the treatment period. Moreover, the activity of SOD was significantly enhanced in the PTC and PC groups relative to the NC group on the 7th day of treatment, while the upward trend continued at 14 and 21 days, observed only in the PC group compared to the NC group. Interestingly, PTC application significantly increased the activity of CAT in comparison to commercial cream (PC group) after three weeks. Additionally, none of the treatments except the PTC treatment made a difference in the level of GSH, showing its enhanced activity. The results are depicted in Figure 8. The antioxidant properties of pine tar can be attributed to its active substances, such as phenols and terpenes in pine tar. Given the similarity in the chemical composition of *Pinus* species, the benefits of accelerated wound healing observed in these species were manifested by a decrease in ROS levels, coupled with a significant increase in GSH and CAT activity, which are consistent with our study.

#### 3.3.5. Histologic Analysis

**Figure 9:**
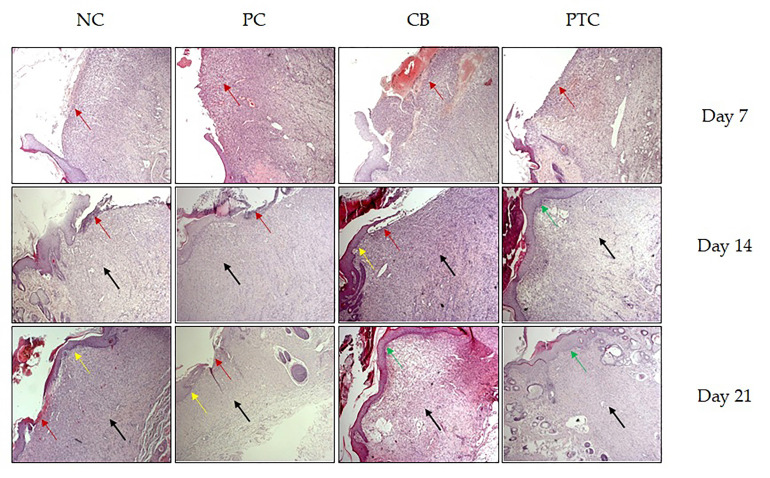
Histological changes in the skin wound section stained with hematoxylin–eosin (H&E). NC—negative control; PC—positive control; CB—cream base; PTC—pine tar cream. Legend arrow: red—necrotic detritus and inflammatory infiltrate; yellow—partial epithelization; green—complete epithelization; black—richly vascularized, more or less, granulation connective tissue. Original magnification 10×.

After seven days of the treatment protocol, we observed the defects, which were covered by necrotic detritus. There was a lower intensity of inflammatory infiltration in the PTC and PC groups compared to the NC and CB groups. Simultaneously, the initiation of capillary blood vessel proliferation was noted, with the most pronounced effect observed in the PTC group. Two weeks later, covering epithelization started in all groups, which was completed in the group treated with PTC. Furthermore, we observed newly formed granulation tissue across all groups. There was a notable presence of intense inflammatory infiltrate in the NC and CB groups, contrasting with the groups treated with pine tar and silver sulfadiazine. Simultaneously, the highest number of newly created capillary blood vessels was found in the NC and CB groups, whereas the most significant reduction was observed in defects treated with PTC. Finally, complete epithelialization was achieved in rats treated with PTC after three weeks of application. On the other hand, in the CB and PC groups, partial epithelialization occurred with remains of necrotic detritus and inflammatory exudate on the surface, with richer subepithelial granulation tissue and easy infiltration by inflammatory cells. In the cream-treated group, there was richly vascularized granulation tissue in the subepithelial area, which was more mature in the pine tar-treated wounds, with a reduced vascular network and scarce inflammatory infiltration. The results are presented in Figure 9.

## 4. Conclusions

- Phytochemical profiling of pine tar revealed the presence of both phenols and terpene compounds, with the most dominant being methyl dehydroabietate, dehydroabietic acid, and reten.
- Physical, chemical, microbiological, and analyses of pine tar cream demonstrated its stability during storage for six months under various temperature conditions. Rheological measurements for pine tar cream suggest easy application, spreadability, and sustained adherence to the skin. These properties are essential for bringing the product to market.
- PTC is safe for skin application since there were no signs of dermal irritation in rats.
- Cream containing 1% pine tar exhibited a notable wound-healing efficacy. The effectiveness was confirmed by a reduction in wound area, enhanced hydroxyproline content, and decreased systemic markers of oxidative stress and histologic analysis.

## Figures and Tables

**Table 1:** Cream base and pine tar formulation.

Ingredient	CB (%) *	PTC (%) *
stearic acid	10.00	10.00
cetyl alcohol	2.00	2.00
cetearyl alcohol	2.00	2.00
polysorbate 80	2.00	2.00
sweet almond oil	4.00	4.00
glycerin	3.00	3.00
phenoxyethanol	0.80	0.80
TEA 10% *	q.s.	q.s.
citric acid	q.s.	q.s.
PT *	/	1.00
distilled water	ad 100	ad 100

* CB—cream base; PTC—pine tar cream; PT—pine tar; TEA—Triethanolamine; q.s.—quantum satis.

**Table 2:** Identified chemical compounds in pine tar.

Peak No	Compound	RT (min) *	%
1	Eugenol, TMS derivative	15.296	0.58
2	Methyl alpha-D-glucofuranoside,4TMS derivative	16.646	0.60
3	Levoglucosan, 3TMS derivative	18.069	1.44
4	Pimara-8(14),15-dien-18-al	22.411	0.42
5	2-Methylanthracene	22.729	0.35
6	4b,8-Dimethyl-2-isopropylphenanthrene, 4b,5,6,7,8,8a,9,10-octahydro-	23.803	1.98
7	Phyllocladene	24.112	0.83
8	18-Norabieta-8,11,13-triene	24.499	3.50
9	9,9-Dimethyl-9-sila-9,10-dihydrophenanthrene	24.829	0.43
10	Phenanthrene, 3,6-dimethyl-	24.996	2.01
11	Podocarpa-6,8,11,13-tetraen-15-oic acid, 13-isopropyl-, methyl ester	25.225	1.48
12	Podocarpa-8,11,13-trien-15-oic acid, methyl ester	26.007	0.78
13	Phenanthrene, 2,3,5-trimethyl-	26.901	1.72
14	Pimaral	27.236	4.71
15	Podocarpa-8,11,13-triene, 13-isopropyl-	27.603	0.71
16	Podocarp-8-en-15-oic acid, 13α-methyl-13-vinyl-, methyl ester	27.734	0.42
17	4-Epidehydroabietol	27.81	0.76
18	Reten (Phenanthrene, 7-isopropyl-1-methyl-)	28.02	10.08
19	Isopimaral	28.189	6.18
20	Methyl sandaracopimarate	28.408	0.82
21	1-Phenanthrenemethanol, 1,2,3,4,4a,4b,5,6,10,10a-decahydro-1,4a-dimethyl-7-(1-methylethyl)-, acetate, [1S-(1α,4aα,4bβ,10aβ)]-	28.559	0.89
22	Isopimaric acid, TMS	28.657	1.29
23	Dehydroabietal	28.937	0.44
24	benzene, 1,1′-(1,2-ethynediyl)bis [2,4-dimethoxy-	29.214	2.97
25	Methyl isopimarate	29.396	0.45
26	Pimaric acid, TMS derivative	29.534	3.59
27	Abietal	29.617	0.91
28	8-Isopropyl-1,3-dimethylphenanthrene	29.695	2.29
29	Methyl dehydroabietate	30.1	22.44
30	Dehydroabietic acid, TMS derivative	30.856	14.59
31	Abietic acid, TMS derivative	31.314	4.23
32	7-Oxodehydroabietic acid, methyl ester	33.761	0.88
Total % of identified compounds			95.50

* RT—retention time.

**Table 3:** Organoleptic characteristics and physical appearance of both formulations over six months at 4 ± 2 °C, 25 ± 2 °C, and 40 ± 2 °C.

Parameters		CB *			PTC *		
		24 h	90 Days	180 Days	24 h	90 Days	180 Days
color	4 ± 2 °C	white	white	white	brown	brown	brown
	25 ± 2 °C	white	white	white	brown	brown	brown
	40 ± 2 °C	white	white	white	brown	brown	brown
odor	4 ± 2 °C	no	no	no	yes	yes	yes
	25 ± 2 °C	no	no	no	yes	yes	yes
	40 ± 2 °C	no	no	no	yes	yes	yes
homogeneity	4 ± 2 °C	NPS	NPS	NPS	NPS	NPS	NPS
	25 ± 2 °C	NPS	NPS	NPS	NPS	NPS	NPS
	40 ± 2 °C	NPS	NPS	NPS	NPS	NPS	NPS
consistency	4 ± 2 °C	semi-solid	semi-solid	semi-solid	semi-solid	semi-solid	semi-solid
	25 ± 2 °C	semi-solid	semi-solid	semi-solid	semi-solid	semi-solid	semi-solid
	40 ± 2 °C	semi-solid	semi-solid	semi-solid	semi-solid	semi-solid	semi-solid

* CB—cream base; PTC—pine tar cream; NPS—no phase separation.

**Table 4:** pH values and electrical conductivity of CB and PTC over 180 days and storage at 4 ± 2 °C, 25 ± 2 °C, and 40 ± 2 °C.

Parameters		CB *			PTC *		
		24 h	90 Days	180 Days	24 h	90 Days	180 Days
pH	4 ± 2 °C	5.58 ± 0.05	5.44 ± 0.08	5.26 ± 0.04	6.05 ± 0.01	6.01 ± 0.04	5.92 ± 0.07
	25 ± 2 °C	5.60 ± 0.07	5.57 ± 0.06	5.45 ± 0.07	6.16 ± 0.04	5.94 ± 0.01	5.89 ± 0.05
	40 ± 2 °C	5.72 ± 0.03	5.68 ± 0.02	5.51 ± 0.06	6.72 ± 0.03	6.59 ± 0.02	6.41 ± 0.04
electrical conductivity (µS/cm)	4 ± 2 °C	71.10 ± 0.05	67.60 ± 0.10	66.30 ± 0.14	72.70 ± 0.07	70.70 ± 0.04	68.90 ± 0.02
	25 ± 2 °C	75.80 ± 0.08	64.50 ± 0.17	62.10 ± 0.09	74.30 ± 0.03	72.10 ± 0.05	67.20 ± 0.04
	40 ± 2 °C	77.60 ± 0.02	69.20 ± 0.13	60.20 ± 0.07	79.10 ± 0.01	71.60 ± 0.04	69.70 ± 0.08

* CB—cream base; PTC—pine tar cream.

## Data Availability

The authors confirm that the data supporting the findings of this study are available within the article.
